# A Simultaneous Test of Synchrony Causal Factors in Muskrat and Mink Fur Returns at Different Scales across Canada

**DOI:** 10.1371/journal.pone.0027766

**Published:** 2011-11-10

**Authors:** Sergio A. Estay, Abraham A. Albornoz, Mauricio Lima, Mark S. Boyce, Nils C. Stenseth

**Affiliations:** 1 Instituto de Ecología y Evolución, Facultad de Ciencias, Universidad Austral de Chile, Valdivia, Chile; 2 Center for Advanced Studies in Ecology and Biodiversity, Pontificia Universidad Católica de Chile, Santiago, Chile; 3 Department of Biological Sciences, University of Alberta, Edmonton, Canada; 4 Centre of Ecological and Evolutionary Synthesis, Department of Biology, University of Oslo, Oslo, Norway; Australian Wildlife Conservancy, Australia

## Abstract

**Background:**

Synchrony among populations has been attributed to three major hypotheses: dispersal, the Moran effect, and trophic-level interactions. Unfortunately, simultaneous testing of these hypotheses demands complete and detailed data, which are scarce for ecological systems.

**Methodology/Principal Findings:**

Hudson's Bay Company data on mink and muskrat fur returns in Canada represent an excellent opportunity to test these hypotheses because of the detailed spatial and temporal data from this predator-prey system. Using structural equation modelling, support for each hypothesis was evaluated at two spatial scales: across Canada and dividing the country into three regions longitudinally. Our results showed that at both scales mink synchrony is a major factor determining muskrat synchrony, supporting the hypothesis of trophic-level interactions, but the influence of winter precipitation synchrony is also important in eastern Canada. Moreover, mink synchrony is influenced principally by winter precipitation synchrony at the level of all Canada (Moran effect), but by distance at regional level, which might suggest some influence of dispersal at this level.

**Discussion/Significance:**

Our result is one of the few reports of synchrony mediated by trophic-level interactions, highlighting the importance of evaluation of scale effects in population synchrony studies.

## Introduction

The first law of geography said: “everything is related to everything else, but near things are more related than distant things” [1]. This law is key for ecological and evolutionary studies dealing with spatially structured data. In population ecology this law takes great relevance when examining synchrony in numerical fluctuations among populations.

As early as 1924, Charles Elton noticed that populations of lemmings seemed to fluctuate in synchrony over a large geographical area [2]; however, this observation was ignored until 1953 when Moran published his theorem about the role of exogenous factors in the synchronization among populations [3]. Yet, only since the 1990s has the problem been extensively studied [4], [5]. Three mechanisms have been proposed as causal mechanisms underlying population synchrony: dispersal or migration, the Moran effect, and trophic-level interactions [4].

Dispersal refers to the movement of individuals among populations. Theoretical experiments have demonstrated the importance of a small exchange of individuals to synchronize two populations sharing a similar feedback structure (endogenous dynamics) [6], [7]. If dispersal is the major factor synchronizing populations, then a negative synchrony-distance (S-D) relationship is expected, but this relationship must be corrected to avoid the spurious effect of a third environmental variable with the same or shorter negative S-D relationship. Unfortunately, it is impossible (both conceptually and mathematically) to eliminate the potential effect of all environmental variables. In this scenario, the best approach is removing the effect of those environmental variables whose influence on the population dynamics of the focal species has been clearly reported. In this way we could be as confident as possible that the estimated influence of dispersal on population synchrony is free of the influence of the environmental variables.

The Moran effect [3] refers to the synchronizing effect that an exogenous factor exerts on the dynamics of populations in a geographical area [5]. The original Moran theorem states that, in absence of dispersal, the synchrony between two populations is equal to the synchrony between their stochastic perturbations if (and this is an important point) these populations share the same feedback structure and the same parameter values. This is an unrealistic assumption because two separated populations are unlikely to have the same parameters values. However, Royama [8] and Hugueny [9] showed, using the same second-order autoregressive model as Moran, that when two populations share the same feedback structure but they have different parameter values the synchrony between populations is not equal, but proportional to the synchrony between the stochastic perturbations. Moreover, Moran proposed his theorem for linear systems and some authors suggested, based on empirical studies and theoretical experiments, poor performance of this hypothesis in non-linear systems [6], [7], [10]–[15]. However, Cazelles & Boudjema [16] extended the result of Moran to phase synchronization in non-linear systems.

Finally, trophic-level interactions can induce synchrony if the focal populations are dynamically dependent on synchronized populations in a lower or higher trophic level [4], [11]. Also, trophic interactions might induce synchrony if the predators are mobile. This hypothesis is supported by evidence from theoretical models [17] and empirical data [18], [19].

Despite this theoretical background, usually it is difficult to use data to distinguish among these three hypotheses [20], [21]. The pattern of synchrony is dependent on local dynamics [8], [9], [11], and a common assumption in theoretical experiments is that populations share the same feedback structure. To differentiate between dispersal and the Moran effect some authors have suggested that when a negative relationship between distance and synchrony is observed then the principal factor acting is dispersal [7], [21]. But environmental variables like climate also can be spatially structured [22], therefore the synchrony among climatic regimes should decrease with distance too [23]. An evaluation of the particular contribution of each component removing the effect of other factors is needed to draw any conclusion about the causes of population synchrony.

**Figure 1:**
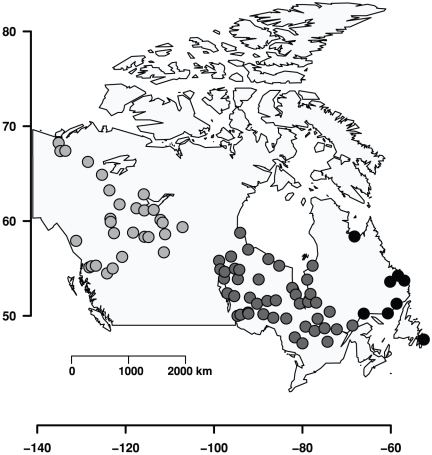
Map of the 81 studied localities in Canada divided in three regions: western Canada (light grey circles), central Canada (dark grey) and eastern Canada (black).

In this context, the annual muskrat (*Ondatra zibethicus* L.) and mink (*Neovison vison* Schreber) fur-returns from the Hudson's Bay Company Archives represent an unique opportunity to evaluate the three mechanisms proposed to explain synchrony among population fluctuations. Data correspond to fur-returns (as a proxy of abundance) at 81 post localities across Canada (Fig. 1). These data together with climatic information for the same localities and the potential predator-prey dynamics observed in the fur returns allow testing of the three mechanisms at the same time. Furthermore, muskrat-mink data have been intensely studied, e.g. [24]–[27] which facilitates the modelling and interpretation of results. In relation to the spatial scale of synchrony in this system, Viljugrein et al. [27] found that synchronization is higher than expected under complete independence between localities for distance up to 540 km, but the strength of phase synchronization increased from west to east [26], although the specific reasons of the observed synchrony remains unknown.

In this study we try to elucidate causal factors of the patterns of synchrony observed in this ecological system. Specifically we evaluated the relative contributions of three hypotheses proposed to explain population synchrony and how the scale of analysis influences this evaluation.

## Results

Models containing summer, autumn and spring climatic variables had a poor performance. They explained 29, 39 and 45% less variance in muskrat synchrony than models containing just winter climatic variables. This is reasonable considering the long duration and harsh conditions during winter across Canada. For these reasons, only those models containing winter variables are shown and discussed.

**Figure 2:**
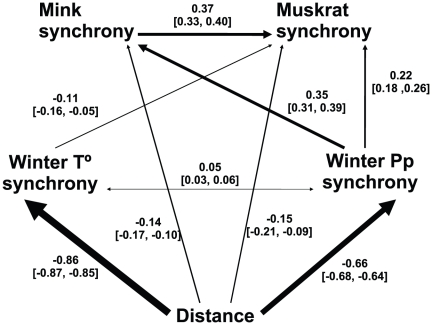
The best structural equation model for all Canada according to BIC criterion. Arrows represent paths. Over each arrow the path value and the confidence interval are shown.

At the level of all Canada, the best model (Table 1, Fig. 2) showed that environmental variables have strong spatial structure. Winter temperature and precipitation synchrony are highly structured spatially according to their path coefficients with distance (−0.86 [−0.85–−0.87] and -0.66 [−0.68–−0.64], respectively). (See Fig. 2, path values and 95% confidence intervals between square brackets).

**Table 1:** Structural equation models for mink and muskrat fur returns from all Canada and each region.

Model	χ2	Adj. GoF	RMSEA	BIC
***All Canada***				
Mink = Dist + Pp + Tm	0.00	1.00	0.00	−8.11
Musk = Dist + Pp + Tm + Mink			[NA ; NA]	
**Mink = Dist + Pp**	**0.10**	**0.99**	**0.00**	**−16.12**
**Musk = Dist + Pp + Tm + Mink**			**[NA ; NA]**	
***Western Canada***				
Mink = Dist + Pp	0.00	1.00	0.00	−6.08
Musk = Dist + Pp + Mink			[NA ; NA]	
**Mink = Dist**	**6.17**	**0.98**	**0.05**	**−12.05**
**Musk = Pp + Mink**			**[NA ; 0.11]**	
***Central Canada***				
Mink = Dist + Pp	0.00	1.00	0.00	−6.90
Musk = Dist + Pp + Mink			[NA ; NA]	
**Mink = Dist + Pp**	**###**	**0.99**	**0.03**	**−10.15**
**Musk = Pp + Mink**			**[NA ; 0.08]**	
***Eastern Canada***				
Mink = Dist + Pp	0.00	1.00	0.00	−3.58
Musk = Dist + Pp + Mink			[NA ; NA]	
Mink ↔ Muskrat	1.54	0.89	0.00	−5.62
Mink = Dist + Pp			[NA ; 0.31]	
Musk = Pp				
Mink = Dist + Pp	0.23	0.98	0.00	−6.94
Musk = Pp + Mink			[NA ; 0.15]	

Each model is described for the path acting on mink synchrony (Mink) and muskrat synchrony (Musk).

Explanatory variables are distance (Dist), winter precipitation synchrony (Pp) and winter temperature synchrony (Tm). Double arrow means correlation. Only models accepted according to χ^2^ criteria are shown. The best models according to BIC are in bold case. Columns include χ^2^ value, adjusted goodness of fit of the covariance matrix (Adj. GoF), root mean square error of approximation (RMSEA), 90% confidence interval for RMSEA (between brackets), and Bayesian Information Criterion (BIC). Due to the method used to get the confidence interval [40] some values appears as NA. This methods sometimes can produce a lower bound above the RMSEA estimate or an upper bound below the estimate; when this happens, the bound is set to NA.

The major influence on muskrat synchrony was mink synchrony (0.37 [0.33–0.40]), and the second was winter precipitation synchrony between localities (0.22 [0.18–0.26]). On the other hand, mink synchrony was influenced principally by winter precipitation synchrony (0.35 [0.31–0.39]; see details in Fig. 2).

**Figure 3:**
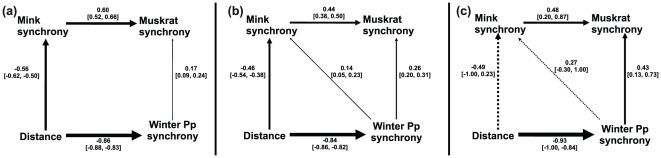
The best structural equation model for each region in Canada according to BIC criterion. Arrows represent path. Solid and dashed arrows are significant and non-significant paths according to 95% confidence interval. Over each arrow the path value and the confidence interval are shown. a) Western Canada, see that the influence of mink synchrony on muskrat synchrony is more than 3 times higher than the influence of winter precipitation synchrony and there is no path from winter precipitation synchrony to mink synchrony. b) central Canada, the influence of mink synchrony is just 1.7 times higher than the influence of winter precipitation synchrony on muskrat synchrony and there is a path from winter precipitation synchrony to mink synchrony. c) eastern Canada, the influence of mink synchrony and winter precipitation synchrony on muskrat synchrony are almost equivalent and the importance of winter precipitation synchrony on mink synchrony increases.

However, in the separate analysis of each region the results are different (see details in Fig. 3). In western Canada, the best model (Table 1, Fig. 3a) showed that mink synchrony continues being the major factor explaining muskrat synchrony (0.60 [0.52–0.66]) and the direct influence of winter precipitation synchrony on muskrat synchrony is small (0.17 [0.09–0.24]). The major factor synchronizing mink is distance (−0.56 [−0.62– −0.50]) rather than winter precipitation synchrony, which is absent in the model. In central Canada, according to the best model (Table 1, Fig. 3b), the influence of mink synchrony is the major factor on muskrat synchrony (0.44 [0.38–0.50]). The second more important factor acting on muskrat synchrony is the influence of winter precipitation (0.26 [0.20–0.31]). Winter precipitation synchrony also showed a small influence on mink synchrony in this region (0.14 [0.05–0.23]). Finally, in eastern Canada, the best model (Table 1, Fig. 3c) maintained mink synchrony as the major factor acting on muskrat synchrony (0.48 [0.28–0.87]), but is similar to the influence of winter precipitation synchrony (0.43 [0.13–0.73]). The path on mink synchrony is not significant in this region, probably due to the small number of localities (n = 8).

In sum our analysis reveals that the apparent causal factors of synchrony change between country and regional scales as well as across a longitudinal gradient is the strength of the mink-muskrat interaction and the influence of winter precipitation synchrony. For example, the effect of mink synchrony on muskrat synchrony is much higher in western than in central and eastern Canada. The inverse relationship appears in the effect of winter precipitation synchrony on muskrat and (but less clear) mink synchrony, which increases from west to east. Finally, distance seems to have influence just on mink synchrony at country and regional scales.

## Discussion

Our analysis reveals clear evidence for scale dependency of the main factor or hypothesis driving the synchrony among populations. Moreover, we emphasize that the three mechanisms potentially responsible for synchrony between populations are not mutually exclusive and they can operate simultaneously. This point is rarely stressed in the literature, but there are no logical reasons to discard the combined operation of these forces as is shown by our analysis (Fig. 3).

Our results show a change in the relative influence of winter precipitation and distance on the synchrony of mink when the geographic scale of the analysis is reduced from country to regional. This phenomenon has been previously shown by Paradis et al. [20]. At the level of all Canada, mink synchrony appears influenced principally by winter precipitation synchrony which suggests that the Moran effect is the more important factor, but at the regional level of analysis the most important path acting on mink synchrony was distance, which adds some relative support for the dispersal hypothesis, given that the effects of the main environmental variables (precipitation and temperature) were removed (see methods). Assuming this, we may suppose that, with a reduction in the size of the study area, dispersal should be more influential because when the distance between populations is short the movement of individuals between them is expected to be higher. On the other hand, at large geographical scales movement of individuals is not homogeneous and climatic phenomena, which act on a greater scale than the maximum dispersal of small mammals, are expected to make a higher contribution to the synchrony among populations.

An interesting result is the magnitude of path values representing Moran effect (paths from temperature and precipitation synchrony to population synchrony in fig. 2 and 3). In a perfect “Moran effect situation” the value of these paths should be close to one. However, even in those models in which Moran-effect paths are the most important, the values of these path are much more lower than one. This result is in agreement with the suggestion of several authors [6]–[10], [14], [15] about the magnitude of Moran effect in populations with different endogenous structure or non-linear structure.

Muskrat synchrony is influenced principally by mink synchrony at the country and regional scales. This result is one of the few empirical examples of synchrony mediated by trophic interactions [18], [19] a result uniquely made possible by the spatial extent of data on both predator and prey.

In the analysis at regional scale we detected several gradients in the estimated path values. However, confidence intervals in the eastern region are 4 to 9-fold wider than in western and central regions. The sample size in this region is small, therefore, there is a lack of power for the estimation of path values reflected by the upper bound in the confidence intervals of RMSEA statistics (exactly 1- the upper bound, [28]). The upper bounds of RMSEA are 0.11, 0.08 and 0.15 for western, central and eastern regions, which suggest that, despite reasonable statistical power in all regions, the confidence in estimated path values in western and central regions is higher than in the eastern region. For this reason we are able to infer differences between western and central regions but not with respect to patterns within the eastern region. In the following paragraphs we will discuss the results in terms of the estimated path values, but it is important to keep in mind that we did not detect differences when the eastern region was involved.

**Figure 4:**
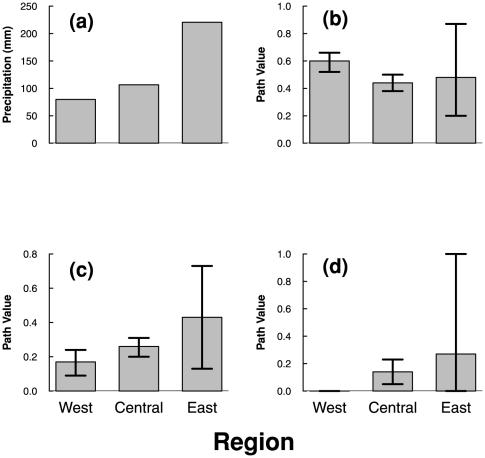
Longitudinal gradients per region in a) total winter precipitation, b) path value of the influence of mink synchrony on muskrat synchrony according to the best model in each region, c) path value of the influence of winter precipitation synchrony on muskrat synchrony according to the best model in each region, and d) path value of the influence of winter precipitation synchrony on mink synchrony according to the best model in each region. In panel d) the lower limit of the confidence interval for the east region was restricted to 0 for graphical reasons, but the real value is −0.30 as can be seen in fig. 3c.

Errington [29] suggested that mink are probably causing the predator-prey cycle and thereby influencing muskrat dynamics, despite being characterized as a generalist predator. In previous studies, the strength of the trophic interaction between muskrat and mink has been suggested to decrease from west to east across Canada. For example, in the eastern region mink and muskrat harvests fluctuate without lag suggesting that minks just follow the fluctuations of muskrats, which can be related to other predators, or both are driven by an exogenous perturbation [27], [30], [31]. These results are in agreement with Erb et al. [24], who showed stronger numerical dependencies between minks and muskrats, higher mink lags and higher prey/predator ratios in western Canada. In our results the path coefficient between mink and muskrat is higher in the west (0.60) than in the central and eastern regions (0.44, 0.48, Fig. 3b and 4b), suggesting that the relative importance of predator-prey interaction is higher in the west than in the central and eastern regions. In fact, a second vein of evidence is given by the second-best model in the eastern region, where mink and muskrat synchrony are just correlated without causal interaction between them (Table 1). One hypothesis suggests that the level of specialization in predation by mink depends on the diversity of alternative prey [25], [31]. For example, Korpimäki et al. [32] found that *Mustela erminea* acts as a specialist predator in northern Fennoscandia, but as a generalist in southern Fennoscandia. Nevertheless, the diversity of predators acting on muskrats could be a reason for the weak predator-prey interaction in eastern Canada too [24], [33].

Another interesting result is the negative relationship between the influence of winter precipitation synchrony on muskrat synchrony and longitude (Fig. 3 and 4c). The influence of winter precipitation on muskrat synchrony in the east is 2.5 times higher than in the west. A similar situation occurs with the influence of winter precipitation on mink synchrony where the best model in the west lacks this path altogether, whereas in the east the path coefficient reaches 0.27 (Fig. 3 and 4d). Errington [29] and Butler [34] suggested several mechanisms through which droughts affect the population level of muskrat: inducing mortality, reducing the habitat available, increasing vulnerability to predation and/or inducing lethal wandering. These mechanisms combined with the suggestion of Errington [29] that predation by mink of muskrat was principally compensatory (on dead and/or weakened muskrats) implies that, if droughts increase vulnerability to predation or the number of unhealthy muskrats, then the rate of predation of mink on muskrats must decrease from west to east in an opposite way to the gradient in precipitation (averages from our data: western Canada  =  79.9 mm, central Canada  =  106.5 mm and eastern Canada  =  220.5 mm). Hence, it is expected that predation exerts a major influence on muskrat population synchrony where precipitation is scarce (western Canada), and winter precipitation synchrony influences muskrat and mink synchrony more strongly in eastern Canada (Fig. 4).

In this study we were able to decipher the importance of climate, dispersal and predation in determining the patterns of synchrony in the mink-muskrat system across Canada. All factors can operate at the same time, but their relative contributions change with scale and geographic location, especially how mink synchrony depends on different factors at country and regional scales, and how the trophic interaction decreases and the influence of winter precipitation increases from western to eastern Canada. Our analysis demonstrated how the three potential mechanisms causing synchrony among populations of mink and muskrat depends on scale and no single mechanism is responsible for synchrony.

## Materials and Methods

### Biological Data

Muskrat and mink 25-years time series from 81 localities across Canada were obtained from the Hudson's Bay Company Archives. Data correspond to fur returns for the period 1925–1949. These data are used as a proxy of abundance and their quality has been previously evaluated [21], [24]–[27], [31]. These authors concluded that fur returns effectively reflects relative abundances, and not a sampling (trapping) artefact., Using an autoregressive model that included price as a predictor variable of harvest, Swanson & Johnson [21] found no relationship between fur price and the harvest of several species including mink and muskrat. On the other hand, Viljugrein et al. [27] compared fur returns with the population scores obtained from questionnaires sent by the Hudson Bay Company to each post (available in the Elton Library, University of Oxford) to evaluate changes in the abundances of several species (including mink and muskrat). They found that scores confirm the observed cycles in fur returns. Finally, Yao et al. [31] grouped the same 81 time series of mink and muskrat used in this study according to their dynamic structure in three clusters, which were approximately coincident with a longitudinal gradient from western to eastern Canada. Data were grouped in this way (Fig. 1) to compare localities with a relatively similar feedback structure, which facilitate, if it exists, the detection of Moran effect [8], [9]. Each region has 29, 44 and 8 localities, respectively.

### Climatic Data

Previous studies suggested that climate could be a key factor in the survival and dispersal of both muskrat and mink in the spring [29], [34], probably due to its influence in the survival of litters and the availability of food and refuges (droughts, ice formation, food, etc.). Mean, minimum and maximum temperatures and total precipitation for each season were considered in the analysis (Winter: December, January, February; Spring: March, April, May; Summer: June, July, August; Autumn: September, October, November). Climatic data were obtained from the historical monthly climate grids for North America [35]. This grid contains the estimated values of monthly precipitation and temperature in the 20th century for United States and Canada at a resolution of 10 km. Estimates were obtained through interpolation using the software ANUSPLIN and real data collected from the National Climatic Data Center (NCDC) in the U.S., and the Meteorological Service of Canada (MSC). Data used in this study correspond to average temperature and total precipitation (rain + snow) from 1925 to 1949.

### Dispersal

Given that a negative relationship S-D is expected [21] under the action of dispersal, we evaluate the effect of dispersal as a direct S-D relationship free of the influence of the best known environmental variables affecting the mink-muskrat system. Evidence suggests that environmental variables with the most important effects are precipitation, snow and temperature, e.g. [29], [34], which are all included in our model. Therefore, we are as confident as possible that the direct influence of dispersal on population synchrony in our model is free of the influence of the environmental variables that could induce spurious results. However, to be cautious in the interpretation, and considering a potential influence of a third spatially autocorrelated environmental variable, hereafter, we refer to this path as a distance effect.

### Modelling

Synchrony between each pair of localities was evaluated using the Pearson product-moment correlation coefficient of the time series of muskrat and mink fur returns. Synchronies between differences in log harvests or rates of change (log *N_t_* – log *N_t_*_-1_) were used to avoid spurious results due to trends [4]. Also synchrony in winter total precipitation (rain + snow) and average winter temperature were calculated to evaluate similarity between each pair of populations and the spatial scale at which these two exogenous forces operate. In this way, we compared matching in the climate regimes (droughts, floods, etc.) across different sites. Finally, dispersal was assumed to be a function geographic distance between each pair of localities after incorporating explicitly the effect of seasonal temperature and precipitation in the model.

To evaluate each hypothesis about synchrony Structural Equations Modelling (hereafter SEM) was used. SEM was developed to quantify the relative contribution of multiple causal paths on a focal phenomenon. These methods, based on the structure of a variance-covariance matrix, are one of the best approximations to study causality in systems with complex interactions and where indirect effects are difficult to distinguish [28], [36].

To proceed with the SEM for all Canada, we constructed a table with 3,321 rows representing all the paired combinations for the 81 localities including the diagonal to fix the intercept to 1, because by definition at distant 0 the correlation must be 1 (*n*×(*n*+1)/2, where *n* is the number of localities), and 5 columns representing each measure of similarity: correlation in muskrat population dynamics, mink population dynamics, winter precipitation regimens, winter temperature regimens and geographic distances. With these data we obtained the correlation matrix which contains the correlation of all paired combinations of variables (5×5 matrix). The key idea here is to use similarities (geographic and between climatic regimens) to explain synchrony (mink and muskrat population dynamics); e.g., path values representing the relationship between the correlation of climatic variables and the correlation of population fluctuations. If the path value is positive, it means that there is a positive relationship between noise correlation and population correlation, as the Moran effect suggests. The same procedure was performed for the three previously defined regions of Canada. Traditional path analysis evaluate the linear dependence between variables (direct and indirect); we evaluated if linear functions were an adequate form to represent the relationship among the used variables (Figures S1, S2, S3, and S4, appendix S1). No difference between squared correlation and linear correlation was higher than 0.1. Moreover, residuals plots show no clear trends and no linear correlation in these plots was higher than 10e(-15), which supports our linear approach (see appendix S1 for details). Moreover, linear modelling is the most parsimonious way to represent the relationship among variables when no information about the particular functional form of the relationship is available and the choice of any other particular form could be difficult to defend.

In this study the correlation matrix was used instead of the variance-covariance matrix to compare the value of each path in the same scale, from −1 to 1 [28], [36]. Each model quantifies paths to test the relative contribution of distance (dispersal), winter climate (Moran effect), and mink synchrony (trophic interactions) on muskrat synchrony. For example, the path value from synchrony in temperature to synchrony in population fluctuations of mink represents how a change in temperature synchrony modifies mink population synchrony. Models were fitted using the sem library [37] in the R environment [38]. All models with an estimated correlation matrix similar to the observed matrix (χ^2^ test) were accepted. This test is exactly equal to the commonly used χ^2^ goodness-of-fit test. Each value in the estimated covariance matrix is compared to the observed value in the real covariance matrix. Because there is not complete independence among each pair of values in the covariance matrix, the degrees of freedom available to test the model are: [*v*(*v*-1)/2]-(*p*+*q*), where *v* is the number of variables, *p* the number of free path coefficients, and *q* the number of free variances of exogenous variables (including the error variables). A non-significant χ^2^ test means that there is no evidence that the observed and estimated matrices differed from each other [28]. Among these models, the best was selected using the Bayesian Information Criterion (BIC or Schwarz Criterion). The model with the smallest BIC was selected as the best model. Because values of synchrony are not completely independent, 95% confidence intervals for each path were estimated by bootstrapping (*n* = 10,000) [4] where a proportional overlap of less of 0.5 could be considered significant [39]. The analysis was performed for all Canada and for each region separately to test for differences due to spatial scale. To avoid spurious results due to high collinearity of climatic variables within each region, regional analyses were performed using only winter precipitation.

## Supporting Information

Figure S1 Testing linearity for variables used in the analysis for all Canada. In the lower triangle, scatterplots between variables. The upper number is the linear correlation coefficient between the variables, the lower number is the quadratic correlation coefficient. In the upper triangle, residual plots after a linear regression between variables. Notice a) No difference between linear and quadratic correlation is higher than 0.1. b) there are no clear patterns in residual plots, which supports our linear approach.
(EPS)

Figure S2 Testing linearity for variables used in the analysis for western Canada. In the lower triangle, scatterplots between variables. The upper number is the linear correlation coefficient between the variables, the lower number is the quadratic correlation coefficient. In the upper triangle, residual plots after a linear regression between variables.
(EPS)

Figure S3 Testing linearity for variables used in the analysis for central Canada. In the lower triangle, scatterplots between variables. The upper number is the linear correlation coefficient between the variables, the lower number is the quadratic correlation coefficient. In the upper triangle, residual plots after a linear regression between variables.
(EPS)

Figure S4 Testing linearity for variables used in the analysis for eastern Canada. In the lower triangle, scatterplots between variables. The upper number is the linear correlation coefficient between the variables, the lower number is the quadratic correlation coefficient. In the upper triangle, residual plots after a linear regression between variables.
(EPS)

Appendix S1 Scale-dependence in the causal factors of synchrony in muskrat and mink fur returns across Canada.
(DOC)
