# Supplementary material for: A Simultaneous Test of Synchrony Causal Factors in Muskrat and Mink Fur Returns at Different Scales across Canada
Source: PLoS One. 2011 Nov 10;6(11):e27766. doi: 10.1371/journal.pone.0027766 (PMC3213188; doi:10.1371/journal.pone.0027766)
Supplement: Appendix S1 — Scale-dependence in the causal factors of synchrony in muskrat and mink fur returns across Canada. (DOC) [file pone.0027766.s005.doc]

**Appendices S1**

**SCALE-DEPENDENCE IN THE CAUSAL FACTORS OF SYNCHRONY IN MUSKRAT AND MINK FUR RETURNS ACROSS CANADA**

**Sergio A. Estay, Abraham A. Albornoz, Mauricio Lima, Mark S. Boyce and Nils C. Stenseth**

**Testing linearity**

Traditional path analysis evaluate the linear dependence between variables (direct and indirect). This linear relationship is the most parsimonious way to represent an univariate statistical association when a priori there is no information about the particular form of the relationship.

In the next figures we show scatterplots of every combination of variables used to calculate the correlation matrix in the path analysis and the residuals of each combinations. Each scatterplot show linear correlation coefficient between variables (cor(x,y)), and quadratic correlation coefficient (cor(x²,y)). No difference between squared correlation and lineal correlation was higher than 0.1. Moreover, residuals plots show no clear trends and no linear correlation in these plots was higher than 10e(-15), which support our linear approach.
